# Neutrophilic inflammation in bronchiectasis

**DOI:** 10.1183/16000617.0179-2024

**Published:** 2025-04-02

**Authors:** James D. Chalmers, Mark Metersky, Stefano Aliberti, Lucy Morgan, Sebastian Fucile, Melanie Lauterio, Patrick P. McDonald

**Affiliations:** 1 Division of Molecular and Clinical Medicine, University of Dundee, Dundee, UK; 2 University of Connecticut School of Medicine, Farmington, CT, USA; 3 Department of Biomedical Sciences, Humanitas University, Milan, Italy; 4 IRCCS Humanitas Research Hospital, Respiratory Unit, Milan, Italy; 5 Department of Respiratory Medicine, Concord Clinical School, University of Sydney, Sydney, Australia; 6 Insmed Incorporated, Bridgewater, NJ, USA

## Abstract

Noncystic fibrosis bronchiectasis, hereafter referred to as bronchiectasis, is a chronic, progressive lung disease that can affect people of all ages. Patients with clinically significant bronchiectasis have chronic cough and sputum production, as well as recurrent respiratory infections, fatigue and impaired health-related quality of life. The pathophysiology of bronchiectasis has been described as a vicious vortex of chronic inflammation, recurring airway infection, impaired mucociliary clearance and progressive lung damage that promotes the development and progression of the disease. This review describes the pivotal role of neutrophil-driven inflammation in the pathogenesis and progression of bronchiectasis. Delayed neutrophil apoptosis and increased necrosis enhance dysregulated inflammation in bronchiectasis and failure to resolve this contributes to chronic, sustained inflammation. The excessive release of neutrophil serine proteases, such as neutrophil elastase, cathepsin G and proteinase 3, promotes a protease–antiprotease imbalance that correlates with increased inflammation in bronchiectasis and contributes to disease progression. While there are currently no licensed therapies to treat bronchiectasis, this review will explore the evolving evidence for neutrophilic inflammation as a novel treatment target with meaningful clinical benefits.

## Shareable abstract

**Neutrophilic inflammation plays a key role in the pathophysiology of bronchiectasis. As there are no licensed therapies to treat bronchiectasis, novel therapies that target neutrophilic inflammation may be beneficial for patients with the disease.** https://bit.ly/3EcbqgK

## Introduction

Bronchiectasis (also sometimes referred to as noncystic fibrosis bronchiectasis) is a chronic and progressive inflammatory lung disease characterised by abnormal dilation of the bronchi accompanied by chronic cough, abnormal sputum production and recurring exacerbations. The pathophysiology of bronchiectasis has been described as a vicious vortex of interlinked components of chronic airway inflammation, recurring airway infection, impaired mucociliary clearance and progressive lung damage [1]. Chronic airway inflammation is a key component in bronchiectasis and neutrophil-mediated inflammation plays a pivotal role in driving the pathogenesis and progression of the disease. This review aims to evaluate the role of neutrophils and neutrophilic inflammation in the pathogenesis of bronchiectasis.

Bronchiectasis is becoming increasingly prevalent worldwide, which may be due in part to improved diagnosis rates and increased recognition of the importance of confirming a diagnosis. Women and those older than 60 years are most commonly affected [2, 3]. Symptoms include chronic cough, sputum production, recurrent respiratory infections, dyspnoea and fatigue [4]. A diagnosis of clinically significant bronchiectasis requires the fulfilment of both clinical and radiological criteria [5]. Radiological criteria for the diagnosis of the disease include dilation of the bronchial wall such that the internal lumen diameter exceeds that of the accompanying pulmonary artery and lack of tapering and visibility of airways in the periphery. Other radiological features of the disease include bronchial wall thickening and mucus plugging, as well as centrilobular nodules and the “tree-in-bud” pattern [4, 6].

Bronchiectasis is very heterogeneous in aetiology and phenotype. According to data from the European Bronchiectasis Registry (EMBARC), bronchiectasis is also highly dependent on patient demographics and characteristics. This study of nearly 17 000 patients in 28 countries in Europe found that the most common aetiology of bronchiectasis was post-infective disease, including tuberculosis (TB). Other associated diseases were COPD, asthma, immunodeficiency, primary ciliary dyskinesia (PCD), allergic bronchopulmonary aspergillosis, connective tissue disease, gastro-oesophageal reflux disease, inflammatory bowel disease and nontuberculous mycobacteria (NTM) infection. In 38% of participants, no cause of bronchiectasis was identified [7]. Whilst similar trends have been reported in the US Bronchiectasis and NTM Research Registry [8] and the Australian Bronchiectasis Registry [9], data from Asia have shown a higher frequency of TB-associated disease [10].

## Pathophysiology of bronchiectasis

**FIGURE 1:**
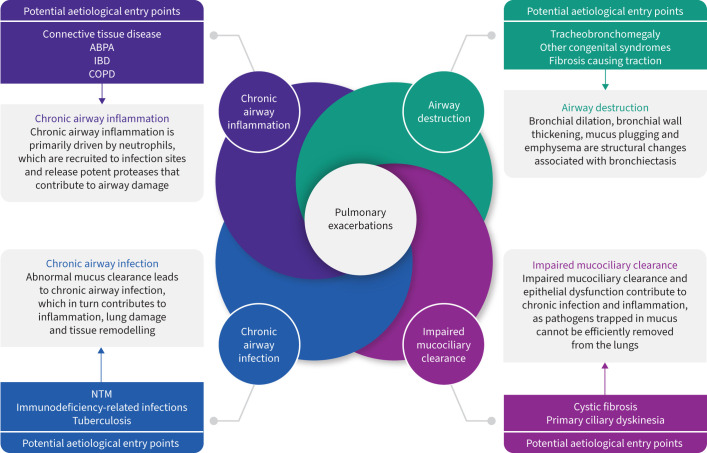
The vicious vortex in bronchiectasis. The vicious vortex in bronchiectasis consists of four components, namely chronic airway inflammation, airway destruction, impaired mucociliary clearance and chronic airway infection. All components of the cycle interact with and influence one another. Each of these components represents a potential aetiological entry point for conditions that can lead to bronchiectasis (*e.g.* allergic bronchopulmonary aspergillosis (ABPA) leading to chronic airway inflammation). IBD: inflammatory bowel disease; NTM: nontuberculous mycobacteria.

Once considered a vicious cycle of ciliary dysfunction and impaired mucociliary clearance, chronic infection, chronic inflammation and lung damage, the pathophysiology of bronchiectasis is now more often referred to as a vicious vortex with interactions between all components of the cycle (figure 1) [1, 11]. Any of these factors, depending on the aetiology, may act as the trigger for the condition. For example, acute or chronic airway infection may be the primary trigger, promoting inflammation, lung damage and mucus hypersecretion, as seen in post-infective disease or NTM infection. Alternatively, abnormal mucociliary clearance, present in conditions such as PCD, results in chronic bacterial infection, inflammation and airway injury and dilation.

Chronic neutrophilic inflammation is a key feature of bronchiectasis pathophysiology and is associated with increased risk of exacerbations and disease progression [12]. Consequently, in the next section, we briefly describe mucociliary clearance, structural damage and infection, before focusing the rest of the review on the role of neutrophilic inflammation.

### Impaired mucociliary clearance

Mucociliary clearance is an important defence mechanism for removing pathogens and pro-inflammatory particles from the lungs. In bronchiectasis, there is a failure of mucociliary clearance believed to be due to the combined effects of ciliary dysfunction, reduced numbers of ciliated cells, excess mucin production (resulting from goblet cell hyperplasia and metaplasia) and mucus dehydration or hyperconcentration. Ramsey *et al.* [13] showed that mucus from patients with bronchiectasis is hyper-concentrated and more viscous and has higher elasticity when compared with induced sputum from healthy individuals. Sputum retention in the airways creates a harbour for infection and mucus obstruction is pro-inflammatory. Airway obstruction can lead to bronchial dilation, which may initiate or lead to progression of bronchiectasis. Both PCD and cystic fibrosis (CF) exemplify the severe phenotype of bronchiectasis that results from profound impairment of ciliary function and mucus hydration, respectively.

### Airway/lung tissue destruction

Bronchiectasis is characterised by structural changes in both the small and large airways. Obstruction of small distal airways leads to dyspnoea, and permanent widening of the bronchial lumen is observed in larger airways [14, 15]. Bronchial dilation, bronchial wall thickening and mucus plugging are common structural changes observed radiologically in bronchiectasis [16]. Airway remodelling is frequently observed in pathological studies and is associated with mucus plugging, which provides an optimal environment for chronic bacterial infection and airway obstruction [17]. Airway obstruction contributes to bronchial dilation and these structural airway defects can, in turn, lead to further impairment of mucociliary clearance [17]. Tissue and cell damage, as part of the attempted healing process, activate inflammatory cells and trigger inflammatory pathways, leading to an increased inflammatory response [18].

### Chronic airway infection

Chronic airway infection with bacteria and, to a lesser extent, infection with fungi or viruses, contributes to the vicious vortex in bronchiectasis [19]. Bacteria, the pathogens most commonly associated with bronchiectasis, can modulate mucin overproduction [20] and promote the release of chemotactic factors that recruit neutrophils to the site of infection [21]. Furthermore, bacteria can secrete toxins and enzymes that can damage lung structure, as well as damage motile cilia and decrease ciliary beat frequency [22]. The two most common pathogens isolated from patients with bronchiectasis are *Pseudomonas aeruginosa* and *Haemophilus influenzae* [22]. NTM [23], *Staphylococcus aureus* [24], *Stenotrophomonas maltophilia* [25], *Moraxella catarrhalis* [26] and *Enterobacteriaceae* [26] are also frequently observed. Viruses are also believed to be important triggers of exacerbation. Rhinovirus has been shown to facilitate secondary bacterial infection by disrupting airway epithelial barrier function [25]. Severe acute respiratory syndrome coronavirus 2 [26], influenza [27] and respiratory syncytial virus [28] can impair mucociliary clearance. Chronic infection drives persistent neutrophilic inflammation. The role of fungi in the development of acute infection and disease course of bronchiectasis remains unclear, with *Aspergillus fumigatus* and *Candida albicans* representing the most common isolated fungi [29].

### Chronic airway inflammation

Neutrophils play a critical role during acute airway inflammation [30]. They are the first white blood cells recruited to sites of infection and can initiate a variety of defence mechanisms, which will be discussed in this review. However, if acute inflammation is not resolved, neutrophilic inflammation persists and contributes to a wide range of chronic diseases, including bronchiectasis. While various cell types, including eosinophils, macrophages, lymphocytes and epithelial cells, are involved in the inflammation observed in bronchiectasis, excessive, uncontrolled neutrophilic inflammation plays a central role in disease progression and is clearly a marker of more severe disease, frequent exacerbations, higher burden of disease and poorer clinical outcomes [12, 31]. Neutrophilic inflammation is associated with increased bacterial load and loss of lung microbiota diversity in patients with bronchiectasis, suggesting that infection is a key driver of inflammation [32]. However, inflammation in bronchiectasis can occur without the presence of infection, such as from exposure to air pollution [33]. Increased levels of neutrophilic inflammatory biomarkers, such as neutrophil elastase (NE), have been associated with worse clinical outcomes in bronchiectasis [34].

## Neutrophil life cycle and function under normal conditions

### Neutrophil generation, maturation and granule formation

**FIGURE 2:**
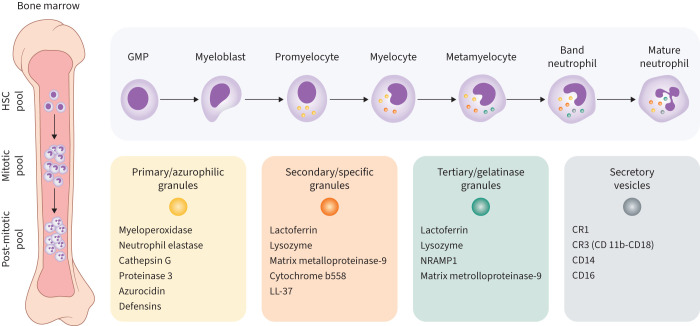
Neutrophil generation, maturation and granule formation. The neutrophil population in the bone marrow consists of the haematopoietic stem cell (HSC) pool, the mitotic pool, and the post-mitotic pool. Haematopoietic stem cells differentiate into granulocyte-macrophage progenitor cells (GMPs), which differentiate into myeloblasts that, in turn, differentiate into neutrophils. As they differentiate, neutrophils gradually acquire different types of granules containing key antimicrobial components. CD11b: integrin alpha M; CD14: cluster of differentiation 14; CD16: Fc-gamma receptor III; CD-18: integrin beta chain-2; CR: complement receptor; NRAMP1: natural resistance-associated macrophage protein 1.

Neutrophils, the most abundant white blood cells in humans, constitute approximately 60% of leukocytes and are the first line of defence in the innate immune system. Neutrophils play a vital role in host defence against bacterial, viral and fungal infections and are produced in the bone marrow from haematopoietic stem cells that differentiate into granulocyte-macrophage progenitor cells. These multipotent progenitor cells eventually differentiate into myeloblasts, committing to the neutrophil lineage [35, 36]. The differentiation process that follows includes myeloblasts turning into promyelocytes and then myelocytes. The latter further differentiate into metamyelocytes and then band cells until, finally, mature neutrophils are formed and released into the circulation (figure 2) [36]. Mature neutrophils contain several types of granules, the formation of which begins between the promyelocyte and myelocyte stages of development [37].

Neutrophil granule proteins have diverse functions, including the destruction of pathogens, the attraction and activation of monocytes and lymphocytes, and enhancing the mobilisation of neutrophils to sites of inflammation by causing tissue damage [38]. Primary or azurophilic granules are generated at the myeloblast-to-promyelocyte stage and store the most toxic compounds, such as elastase, myeloperoxidase (MPO), cathepsins and defensins [37, 39]. These function primarily within the phagolysosome and have antibacterial properties. Serine proteases within these granules also have the ability to degrade extracellular matrix (ECM) proteins and aid pathogen digestion and clearance [40]. Secondary or specific granules are formed at the myelocyte and metamyelocyte stages and contain antimicrobial proteins such as lactoferrin, matrix metalloproteinase (MMP)-9, and NADPH (nicotinamide adenine dinucleotide phosphate) oxidase subunits. Tertiary or gelatinase granules are formed at the band cell stage and their content partially overlaps that of secondary granules. Secretory vesicles are detected only in mature neutrophils and are the most easily mobilised [39, 41, 42]. Their membranes contain various chemoattractant and phagocytic receptors, as well as adhesion molecules associated with enhanced chemotaxis and/or phagocytosis [43]. The separation of granules into different subsets is somewhat arbitrary, as granules represent a continuum between denser, older, harder-to-mobilise granules and lighter, more recently formed, easier-to-mobilise vesicles. This is also why granule contents often overlap between two or more subsets [44, 45].

Serine proteases account for more than one-third of known proteolytic enzymes [46]; among them, neutrophil serine proteases (NSPs), which originate in the primary granules, play an important role in host defence, including pathogen killing. NSPs are synthesised as inactive proteins and require post-translational cleavage to be activated and packaged into granules. The main NSPs are NE, cathepsin G (CatG) and proteinase 3 (PR3), which are activated during neutrophil maturation in the bone marrow by dipeptidyl peptidase 1 (DPP1), also known as cathepsin C [47, 48]. These proteases can kill bacteria within the neutrophil after phagocytosis or *via* extracellular release following degranulation; NSPs may also kill bacteria that are ensnared within neutrophil extracellular traps (NETs) [49]. Patients with the rare genetic disease Papillon–Lefèvre syndrome (PLS) have loss of function mutations in the *CTSC* gene, which codes for DPP1 [50]. Their neutrophils lack active NSPs and are unable to form NETs in response to exogenous reactive oxygen species (ROS) or phorbol-12-myristate-13-acetate, but can still kill bacteria *via* phagocytosis. Importantly, these patients have no marked immunodeficiency [50–53].

### Neutrophil effector functions

**FIGURE 3:**
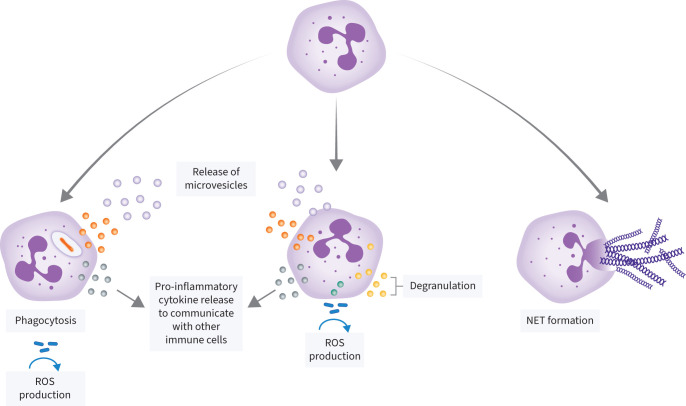
Neutrophil effector functions. Neutrophils control and eliminate pathogens *via* various mechanisms, including phagocytosis, degranulation and reactive oxygen species (ROS) generation, the formation of neutrophil extracellular traps (NETs), microvesicle production and cytokine release.

Neutrophils control and eliminate pathogens *via* three key mechanisms, namely phagocytosis, degranulation and ROS generation, and the release of NETs (figure 3). Neutrophils can engulf bacterial and fungal pathogens, as well as dead cells and tissue debris, *via* phagocytosis. Oxygen-independent and -dependent processes are used to kill ingested micro-organisms. Oxygen-independent processes employ a variety of lytic enzymes and antimicrobial proteins, whereas oxygen-dependent mechanisms mainly involve potent antimicrobial ROS such as superoxide, hydrogen peroxide and hypochlorous acid [54–56]. During degranulation, neutrophil granules fuse to the cell membrane, which triggers the release of their antimicrobial contents into the extracellular space. Concurrently, the NADPH oxidase complex assembles at the cell membrane, allowing for the extracellular generation of ROS [57]. Additionally, neutrophils can produce microvesicles, which may be capable of restricting bacterial growth and dissemination [58]. NETs are weblike structures made of extruded, decondensed chromatin released by neutrophils. They contain numerous cytosolic and granular proteins, including antimicrobial peptides and proteases. NETs immobilise invading pathogens, preventing their dissemination and contributing to their destruction. NET formation usually entails cell death, a process referred to as NETosis [59]. However, some studies have demonstrated a process known as vital NETosis, in which NET formation does not require neutrophil cell death [60–62]. Neutrophils also express and produce pro-inflammatory cytokines and chemokines [63, 64]. Some of the chemokines produced by neutrophils are primarily chemotactic for neutrophils themselves (C-X-C motif chemokine ligand (CXCL) 1, CXCL8, CXCL9), while other neutrophil-derived chemokines attract immune cell types such as monocytes, dendritic cells, B-cells and T-cells [63]. Collectively, these chemokines contribute to the sequential recruitment of distinct leukocyte populations into inflamed tissue. Cytokines produced by neutrophils modulate the immune response and the expression of C-C chemokine receptor 7 by activated neutrophils promotes their migration to lymph nodes, where they can act as antigen-presenting cells [65].

### Neutrophil apoptosis and efferocytosis

**FIGURE 4:**
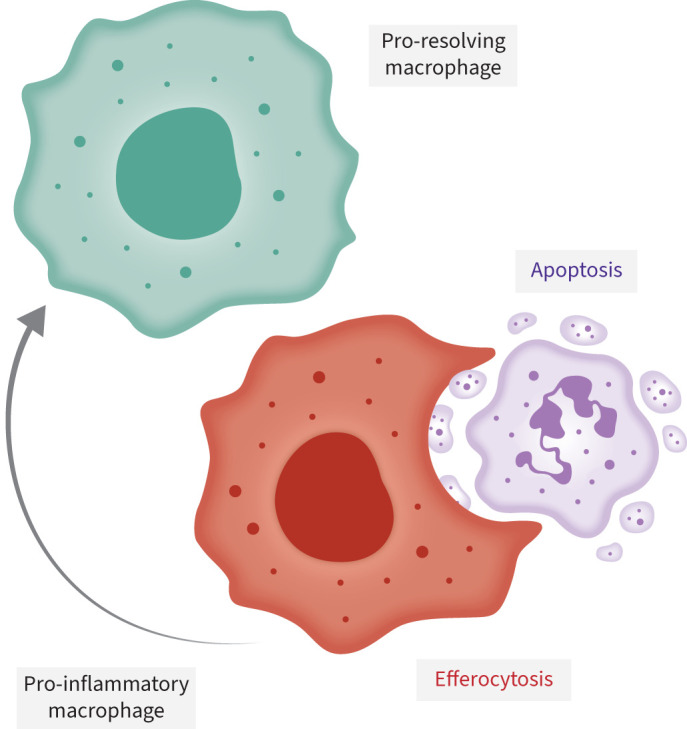
Neutrophil efferocytosis. Efferocytosis involves the removal of apoptotic cells by phagocytes such as macrophages.

Neutrophils are short-lived cells with a half-life in blood of only 6–10 h. However, their life span greatly increases once they have migrated into tissues, as anti-apoptotic molecules are upregulated in activated neutrophils [66]. The life span of neutrophils in different tissues and under different inflammatory states is poorly defined [67]; however, it has been estimated that the life span of the neutrophil may increase up to 7 days in tissues in response to inflammatory or infectious agents [68]. The removal of apoptotic cells, called efferocytosis, involves their uptake by other phagocytes, such as macrophages or dendritic cells, a process that is critical for resolution of acute inflammation (figure 4) [69, 70]. This is especially crucial in the case of neutrophils as they would otherwise lyse and release their immunogenic and toxic contents extracellularly, resulting in further inflammation and tissue damage. Macrophages in the liver and spleen, in direct contact with the blood, are involved in the clearance of circulating apoptotic or senescent neutrophils [71]. Upon phagocytosis of apoptotic neutrophils, pro-inflammatory macrophages cease to produce pro-inflammatory cytokines as they become pro-resolving macrophages, thereby promoting a return to homeostasis. Pro-resolving macrophages produce anti-inflammatory cytokines and mediators, including transforming growth factor-β1 and prostaglandin E2 [72].

## Dysregulation of neutrophilic inflammation in bronchiectasis

Acute airway inflammation is a transient and self-limiting defence mechanism in response to pathogens, injury and toxins, which aims to protect the lungs by removing noxious stimuli and restoring homeostasis [73]. Airway inflammation should be resolved upon the clearance of infection and sufficient repair of tissue. Chronic inflammation may occur if resolution is ineffective or incomplete, resulting in leukocytes such as neutrophils continually being recruited to the site of inflammation and further driving inflammation [74].

**FIGURE 5:**
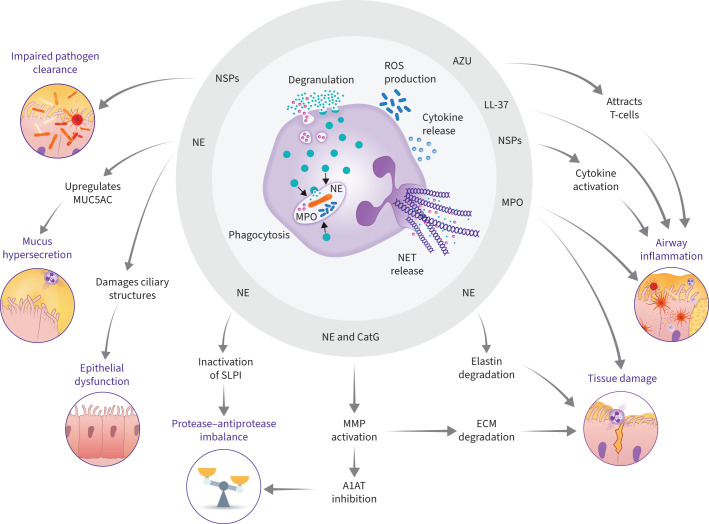
An overview of the downstream effects of neutrophilic pro-inflammatory mediators. Neutrophil effector functions result in the release of various neutrophil-derived mediators and potent proteases such as LL-37, myeloperoxidase (MPO), azurocidin (AZU) and neutrophil serine proteases (NSPs). LL-37 and MPO can contribute to tissue damage in the lungs. AZU acts as a chemoattractant for T-cells and aids epithelial dysfunction. NSPs contribute to the protease–antiprotease imbalance observed in bronchiectasis by cleaving and inactivating protease inhibitors such as secretory leukocyte protease inhibitor (SLPI) and alpha-1 antitrypsin (A1AT). NSPs activate matrix metalloproteinases (MMPs), which can also inhibit antiproteases such as A1AT, as well contribute to the destruction of the extracellular matrix (ECM). High, uncontrolled NSP levels can impair pathogen clearance in the lungs, resulting in chronic infection. NSPs also promote the generation of inflammatory effectors such as cytokines and chemokines, which amplify inflammation. NSPs also contribute to epithelial dysfunction by decreasing ciliary motility and damaging ciliary structures. Neutrophil elastase (NE) contributes to ECM destruction and remodelling by degrading elastin fibres as well as collagen and laminin. NE increases the expression of the gel-forming mucin 5AC (MUC5AC), which leads to mucus hypersecretion. CatG: cathepsin G; ROS: reactive oxygen species.

Chronic neutrophilic inflammation is a central feature of bronchiectasis; failure to resolve this inflammation contributes to the bronchiectasis vicious vortex. Inflammation in bronchiectasis involves the release of potent serine proteases and other inflammatory mediators from neutrophils that damage lung tissue and impair host defences (figure 5) [75]. The driving force behind this persistent inflammation is not completely understood, but it is believed that protease–antiprotease imbalance is a contributory factor [49]. Patients with bronchiectasis have higher sputum neutrophil counts [76], which correlate with disease severity [31, 77]. Patients with more severe disease have higher blood neutrophil counts, which correlate with both impaired lung function and disease severity [78]. In addition, sputum NET concentration has been shown to be higher in patients with bronchiectasis *versus* healthy controls and correlates with disease severity and risk of exacerbation [31]. There is also evidence to suggest that blood neutrophils are reprogrammed in bronchiectasis and have prolonged survival, impaired neutrophil phagocytosis and impaired killing of *P. aeruginosa* [79].

### NSPs and their role in bronchiectasis inflammation

Although NSPs have an important function in host defence, they are also involved in the pathogenesis and destruction of lung tissue in bronchiectasis. A prospective, observational study from the UK found that elevated sputum NE activity correlated with disease severity and exacerbations [12]. In that study, markers of disease severity included bronchiectasis severity index (BSI), Medical Research Council dyspnoea score and St George's Respiratory Questionnaire (SGRQ), and NE levels were associated with all three markers. The severity of radiological bronchiectasis was determined using the Reiff score and increased NE was associated with increased radiological severity. Additionally, an association between spirometry values such as absolute forced expiratory volume (FEV) and the predicted percentage for FEV in 1 s (FEV_1_ %) was observed. NE correlated with microbiological data in these patients. Increased NE activity was associated with increased airway bacterial load (at bacterial load >10^7^ CFU·mL^−1^). Furthermore, higher levels of NE were observed in patients chronically infected with *P. aeruginosa*, *Enterobacteriaceae* and *H. influenzae*, compared with patients without chronic infection. Sputum NE activity increased during exacerbation and, after a 3-year follow-up, higher sputum NE activity was associated with higher frequency of exacerbations. Moreover, sputum NE was responsive to antibiotic therapy and failure to return to baseline NE levels after treatment was associated with shorter time to next exacerbation. NE contributes directly to lung destruction by degrading elastin, a protein that provides elasticity to the lungs. In this same study, serum desmosine, a degradation product of elastin [80], was also found to have a weak correlation with sputum NE and was associated with an increased risk of severe exacerbation [12]. These data were partially replicated in two Southern European cohorts of patients with bronchiectasis. This study found that sputum NE activity correlated with disease severity, lung function, chronic infection and quality of life (QoL) [34].

NE has been shown to upregulate epithelial Na^+^ channel activity, which may lead to increased Na^+^ absorption, further dehydration of the airway surface liquid and obstruction [81]. NE trans-activates epidermal growth factor receptor (EGFR), a host cell surface protein that plays a role in sustaining neutrophilic inflammation [82]. Conversely, NE can disrupt the alveolar epithelial barrier *via* the cleavage of EGFR [83]. Additionally, NE promotes degradation of the cystic fibrosis transmembrane conductance regulator, an additional mechanism whereby NE contributes to mucus hyperconcentration [84]. NE mediates apoptosis of epithelial cells, which contributes to lung injury [85] and increases the expression of mucin 5AC (MUC5AC) in respiratory epithelial cells [86], and induces goblet cell metaplasia, which results in mucus hypersecretion [87]. NE can decrease mucociliary function and ciliary beat frequency *in vitro* [88]. Furthermore, NE has been shown to correlate with low microbiome diversity and *P. aeruginosa* infection in patients with bronchiectasis [89].

NSPs also play a role in controlling local immune responses by proteolytic regulation. This has been demonstrated by the presence of secretory leukocyte peptidase inhibitor (SLPI) cleavage fragments in lower airway secretions of patients with bronchiectasis with high MMP-9 and NE levels [90]. SLPI is a low molecular weight, cationic protein with antiprotease and antimicrobial activity that strongly inhibits NE, as well as CatG, trypsin and chymase, and protects tissues from damage by these proteases during inflammation. SLPI inhibits NE-mediated degradation of fibronectin [91]; however, NE-mediated cleavage of SLPI further contributes to damage and inflammation. Cleaved SLPI has a significantly lower ability to bind lipopolysaccharides and a reduced capacity to inhibit NE activity [90]. Additionally, NE reduces the secretion of SLPI from epithelial cells in the lungs [92]. SLPI inactivation may be followed by increased proteolysis, tissue damage and inflammation [90]. Reduced SLPI levels are associated with greater exacerbation frequency as well as *P. aeruginosa* infection [93]. NE can also cleave several opsonins involved in innate immunity, such as surfactant proteins D [94] and A [95], as well as immunoglobulins [96], complement components and cell surface receptors in neutrophils [97].

A murine model of endobronchial inflammation demonstrated that CatG inhibited the host's ability to clear *P. aeruginosa* from the lungs. These findings were based on a 1-log reduction in bacteria recovered from CatG-deficient mice and suggest that CatG plays a role in impaired microbial clearance in bronchiectasis [98]. NE and CatG can both activate MMPs [99, 100], which contribute to ECM degradation and inactivation of alpha-1 antitrypsin (A1AT) [101], and older studies have demonstrated a role for CatG in the destruction of the airway epithelium [102]. CatG activity is higher in patients with bronchiectasis than in healthy controls and this activity increases with disease severity [103].

During bronchiectasis exacerbations, PR3 is elevated in sputum and the EMBARC exacerbation cohort study found it was significantly higher in patients with chronic *P. aeruginosa* infection and was associated with other clinical and radiological markers of active lung inflammation and infection [104, 105].

### Other neutrophil-derived products reported to contribute to inflammation in bronchiectasis

#### Azurocidin (AZU)

Human neutrophils express AZU, an NSP homologue that is catalytically inactive but has potent antimicrobial properties, can act as a chemoattractant for T-cells [106] and promotes endothelial and epithelial dysfunction. AZU has been shown to have a marked effect on ciliated epithelial cells by reducing ciliary beat frequency, which may directly influence risk of infection [107]. AZU has potential as a marker of airway inflammation and disease severity in bronchiectasis and correlates with NE levels in sputum [108].

#### MMPs

MMPs are endopeptidases that are primarily involved in ECM remodelling. However, these diverse enzymes are involved in other immunological processes such as modulating cytokine and chemokine activity, opening a path through the ECM for cell migration, regulating leukocyte recruitment and activating defensins [109]. MMP-9 is secreted by neutrophils, macrophages and fibroblasts and is inhibited by the tissue inhibitors of MMPs (TIMPs). Evidence suggests that some MMPs, including MMP-9, are involved in the pathophysiology of bronchiectasis. For example, Taylor *et al*. [110] found that increased levels of MMP-9, as well as MMP-8 and increased MMP:TIMP ratios, correlated with decreased lung function and higher levels of inflammatory markers in patients with bronchiectasis. In the same study, whereas levels of MMP-8 and MMP-9 were increased in bronchiectasis, their primary inhibitor TIMP-1 was not, indicating a protease-antiprotease imbalance. Other research has revealed that MMP-8 and MMP-9 are associated with lobes harbouring *P. aeruginosa* [111]*.* MMP-8 and MMP-9 are significantly increased in patients with bronchiectasis compared with healthy controls and significantly increased during bronchiectasis exacerbations [112]. Pro-inflammatory cytokines can upregulate the expression of MMPs and drive tissue damage [113].

#### MPO

MPO, a neutrophil product that gives sputum its green colour, is increased during exacerbations [114]. Accordingly, sputum colour is a readily available, qualitative marker of lung infection in clinical practice for patients with bronchiectasis. The Sputum Colour Chart developed by Murray *et al.* [115] uses three major grades of colour, which correlate with bacterial colonisation, as follows: mucoid (clear), mucopurulent (pale yellow/pale green), and purulent (dark yellow/dark green). MPO acts as a catalyst in the formation of some reactive oxygen intermediates, mainly hypochlorous acid [116], which have potent antimicrobial properties but can also damage host tissues and contribute to inflammation. One study found that MPO can cause cellular damage to alveolar and bronchial epithelial cells, as these cells internalise MPO, which can induce DNA strand breakage [117]. MPO-derived hypochlorous acid inactivates TIMP-1 by oxidising its N-terminal cysteine, reducing its ability to inhibit MMPs [118].

#### LL-37

LL-37 is an antimicrobial protein that is released from neutrophils during degranulation and necrotic death and is highly present in NETs. It is produced *via* proteolytic cleavage of the hCAP18 (human cathelicidin antimicrobial protein 18) precursor protein by PR3 [119]. This protein may modulate inflammation by acting as a chemoattractant for both neutrophils and eosinophils [120]. One study found that elevated LL-37 and reduced SLPI levels were associated with *P. aeruginosa* infection, higher disease severity and increased risk of future exacerbations [93]. Furthermore, the same study established that elevated LL-37 and reduced SLPI levels were associated with the frequent exacerbator phenotype in bronchiectasis.

### Neutrophil-associated biomarkers and correlation with disease severity

Biomarkers can aid in the diagnosis of disease, guide treatment and predict outcomes and disease severity. However, bronchiectasis is such a heterogeneous disease that few biomarkers have been identified as being generally useful [121]. Neutrophil-associated biomarkers may prove valuable in predicting disease severity; however, the biomarkers identified are often used in research settings but generally not in clinical practice.

In a study by Goeminne *et al*. [76], compared with healthy controls, patients with bronchiectasis were reported to have higher sputum neutrophil and NE levels, as well as increased MMP-9 levels, when chronic *P. aeruginosa* infection was detected [77]. Additionally, in the same study, the authors demonstrated an association between the Sputum Colour Chart and increased markers of inflammation and proteolytic enzymes [76]. Recent data from EMBARC have shown that, during a 5-year follow-up period, sputum colour at baseline was strongly predictive of future bronchiectasis exacerbations. The risk of mortality also significantly increased with increasing sputum purulence. The authors proposed that sputum colour may represent a simple biomarker that could be used to predict outcomes [122]. The results of these studies correlate well with previous research that demonstrated links between sputum colour and bacterial infection [115] and activity of markers of bronchial inflammation such as NE and MPO [123].

Malondialdehyde (MDA) is a marker of oxidative stress. Exhaled breath condensate MDA and sputum neutrophilia correlate with disease severity in patients with bronchiectasis, as confirmed by pulmonary function, disease duration, bacterial colonisation, BSI score and exacerbation rate [77]. Neutrophil side fluorescence can reflect neutrophil activation state and may be a predictive marker of severity in bronchiectasis [124].

Furthermore, sputum NE activity may act as a marker of disease severity and of future risk of disease progression [12]. A novel, semiquantitative, lateral flow, point-of-care device (neutrophil elastase airway test stick or NEATstik) has been shown to identify patients with bronchiectasis at increased risk of airway infection and future exacerbations [125]. In this study, higher NE levels were associated with bronchiectasis severity, a significant increase in exacerbation frequency and shorter time until next exacerbation. However, the NEATstik is not widely used in clinical practice. Sputum NE has also been shown to be a useful marker of bronchiectasis severity in paediatric patients. Sputum NE correlated with frequency of exacerbations, exacerbation severity and hospital admission in patients younger than 18 years [126].

An international multicohort study investigated the relationship between NETs and disease severity and treatment response in bronchiectasis. Sputum NETs or NET components correlated with BSI, QoL (measured using the QoL Bronchiectasis Respiratory Symptom score), future risk of hospital admission and mortality. Furthermore, clinical response to intravenous antibiotic treatment was associated with successfully reducing NETs in sputum [31]. Additionally, elevated airway concentrations of pregnancy zone protein, a component released into NETs, were associated with disease severity, frequent exacerbations and airway infection in patients with bronchiectasis [127].

## The role of other cell types in the pathophysiology of bronchiectasis

Bronchiectasis is considered a primarily neutrophil-driven disease. However, emerging evidence suggests that other cells are also involved in disease progression and may contribute to important disease manifestations such as exacerbations.

### Eosinophils

Eosinophils play a useful role in combating parasitic infections and certain cancers and may be protective in bronchiectasis. They are well equipped to combat common pathogens associated with bronchiectasis *via* the production of proinflammatory cytokines and peptides, such as eosinophil cationic protein, major basic protein and eosinophil peroxidase [128]. Similar to the formation of NETs by neutrophils, eosinophils can also release mitochondrial DNA-containing traps to kill bacteria [129]. Moreover, eosinophils possess antiviral properties and can kill invading viruses *via* degranulation, cytokine production and the generation of superoxide and nitric oxide free radicals [130]. However, despite these protective qualities, eosinophils can also contribute to the pathogenesis of bronchiectasis. Eosinophilic type 2 inflammation in the airways is a characteristic of one phenotype of bronchiectasis associated with asthma. Type 2 inflammation in bronchiectasis with peripheral and airway eosinophilia in the absence of asthma has recently been described. In an analysis of 1007 patients with bronchiectasis from five countries, “eosinophilic bronchiectasis” was defined by elevated eosinophil counts (≥300 cells·µL^−1^) in peripheral blood and the absence of asthma. This subtype affected approximately 20% of patients. Raised blood eosinophil counts were associated with *Pseudomonas* and *Streptococcus* microbiome profiles as well as shortened time to exacerbation (after accounting for infection status) [131]. In other studies, patients with bronchiectasis and eosinophilia had higher levels of fractional exhaled nitric oxide, greater bronchodilator reversibility and higher sputum interleukin (IL) 13 [132, 133]. Eosinophil protease production requires more research; however, eosinophils were recently found to express a unique serine protease, PRSS33. This protease was found to induce fibroblast extracellular matrix protein synthesis, which sheds some light on the role of eosinophils in airway remodelling [134]. Eosinophilic inflammation does not exclude a neutrophilic inflammatory pattern and previous studies have shown that both types of inflammation may coexist in bronchiectasis [132, 135]. The presence of eosinophilia is considered a “treatable trait” for some patients with bronchiectasis and should be routinely assessed.

### Macrophages

Macrophages are also involved in the inflammatory process in bronchiectasis. Inflammatory infiltrate, consisting primarily of macrophages and lymphocytes, obstructs small airways in bronchiectasis [136]. A study analysing endobronchial biopsies of 14 patients with stable bronchiectasis found that patients who were regular sputum producers had significantly higher macrophage density, but not neutrophil density, than those who were not [137].

The clearance of apoptotic cells by macrophages is necessary for the resolution of inflammation. In a small study of children with protracted bacterial bronchitis (PBB) or bronchiectasis, macrophage phagocytic capacity for apoptotic cells was significantly lower in children with PBB or bronchiectasis, compared with healthy controls. Furthermore, the expression of the mannose receptor, usually found on the surface of macrophages, was also significantly reduced in the children with bronchiectasis [138].

Macrophage-stimulating protein (MSP) promotes ciliary motility on the airway ciliated epithelium. A small study investigating the functional involvement of MSP in bronchiectasis found that MSP concentrations were significantly elevated in sputum from patients with bronchiectasis compared with healthy controls. These data suggest that increased MSP may compensate for impaired mucociliary clearance in bronchiectasis [139].

### Lymphocytes

Evidence suggests that B-cell and T-cell dysfunction may be a risk factor in developing bronchiectasis. Increased infiltration of CD3^+^ T-lymphocytes, predominantly CD4^+^ T-cells, and higher levels of CD68^+^ macrophages have been shown in the airways of patients with bronchiectasis compared with healthy controls [140]. Activated phosphoinositide 3-kinase delta syndrome is associated with B-cell and T-cell dysfunction and is characterised by recurrent airway infection and bronchiectasis [140].

Another study found that T-cell responses against *H. influenzae* showed a negative correlation with BSI and a positive correlation with lung function in patients with bronchiectasis. This suggests that T-cells may offer protective immunity against *H. influenzae* and that patients with impaired T-cell responses may have more severe disease [141].

Bronchiectasis is associated with elevated levels of pro-inflammatory cytokines and chemokines such as IL-8, IL-6 and tumour necrosis factor-α (TNF-α) in bronchoalveolar lavage fluid (BALF) [142]. The IL-23/T-helper 17 (Th17) immune response plays an important role in chronic inflammation in bronchiectasis. Th17 cells are a unique CD4^+^ T-cell subset and contribute to the production of inflammatory cytokines and recruitment of leukocytes, especially neutrophils [143]. Th17 cells produce the inflammatory cytokines IL-17A, IL-17F, IL-21, IL-22, TNF-α and IL-10. The IL-23 cytokine regulates the conversion of Th17 cells to pathogenic effectors of chronic inflammation [144]. Chen *et al.* [145] demonstrated significant airway luminal activation of the Th17 pathway in patients with bronchiectasis. Th17 pathway cytokines were quantified in BALF and all Th17 cytokines were significantly higher in patients with bronchiectasis than in healthy controls. Furthermore, IL-1β and IL-8 gene expression in bronchiectasis endobronchial biopsies was significantly higher than in control biopsies. BALF IL-8 and IL-1α levels likewise showed significant relationships with clinical measures and airway microbiology [144].

### Epithelial cells

The airway epithelium uses several mechanisms to defend against pathogens, such as the removal of mucus and potentially harmful substances *via* mucociliary clearance, the secretion of antimicrobial proteins and the release of inflammatory mediators. These mediators lead to the activation and recruitment of neutrophils and other inflammatory cells. In patients with bronchiectasis, TNF-α in bronchial secretions has been shown to enhance the expression of intercellular cell adhesion molecule-1 in bronchial epithelial cells, which may contribute to sustained neutrophil recruitment to the airways [146]. Atypical proliferation of airway progenitor cells in the epithelium of dilated bronchioles has been demonstrated in patients with bronchiectasis, compared with healthy controls, which the authors hypothesised may contribute to the failure of airway injury repair [147]. Epithelial cells express endothelin 1 (ET-1), which promotes neutrophil adhesion to epithelium, the migration of neutrophils to sites of inflammation and the release of elastase. Research has shown that patients with bronchiectasis and *P. aeruginosa* in their sputum had significantly higher serum levels of ET-1 than those who did not and that serum ET-1 levels correlated with 24-hour sputum volume [148].

## Translation to clinical practice

### Identification of clinical phenotypes and endotypes associated with inflammation

A hallmark of bronchiectasis is its heterogeneity. Better identification and diagnosis of clinical phenotypes and endotypes within bronchiectasis, associated with inflammation, may lead to more personalised treatments for patients and allow for better, more targeted recruitment of patients to clinical trials. Various definitions of clinical phenotypes have been proposed according to cause, microbiology, frequency of exacerbations, radiological patterns and symptoms [149–151]. Furthermore, it is now possible to integrate and analyse extensive biological data using multi-omics approaches to better describe patients according to endotypes, defined by a distinct functional or pathobiological mechanism. A deeper understanding of bronchiectasis genetics, as well as the inflammatory pathways involved and the microbiome, may assist in the classification of bronchiectasis endotypes and facilitate this more personalised treatment approach [149].

### Phenotypes

The frequent-exacerbator phenotype is an excellent example of a clinical phenotype. These patients are readily detectable in clinical practice by recording their frequency of exacerbations. Greater exacerbation frequency has been linked to increased disease severity and poor QoL [152]. Patients with more frequent exacerbations, particularly those with *P. aeruginosa,* have increased mortality rates [153]. In paediatric patients, multiple exacerbations are linked to younger onset of disease, *P. aeruginosa* infection and previous hospitalisation [154].

Patients with NTM infection often have distinct clinical characteristics, being mostly post-menopausal women, and are reported to have distinguishing morphological traits [155]. The post-TB bronchiectasis phenotype was associated with higher rates of haemoptysis, NTM isolation, lower FEV_1_, more frequent exacerbations and greater disease severity [156], as well as reduced lung function and more frequent use of both inhaled bronchodilators and mucolytics [157].

The COPD–bronchiectasis overlap phenotype, which defines patients with both COPD and bronchiectasis, could be a clinical predictor of response to treatment [158]. Research has shown that this phenotype, compared with COPD without bronchiectasis, is linked to increased risk of daily sputum production, exacerbation, more frequent hospital admissions and a higher isolation rate of *P. aeruginosa* [159].

The asthma–bronchiectasis overlap phenotype suggests that early identification of bronchiectasis in patients with severe asthma is crucial to improve treatment and clinical outcomes [160].

### Inflammatory endotypes

Neutrophilic inflammation is the predominant inflammatory endotype in bronchiectasis. However, a distinct eosinophilic endotype has been identified, characterised by higher exacerbation rates and increased disease severity [161]. Notably, eosinophilia can coexist with neutrophilic inflammation in a subset of patients and does not indicate the absence of neutrophilic inflammation [133, 162].

Distinct inflammatory endotypes were identified in an analysis of 199 patients with stable bronchiectasis enrolled at three European centres in the EMBARC-BRIDGE study [135]. Cluster analysis was used to identify and validate endotypes according to their inflammatory profiles. Four clusters were identified, as follows: cluster 1 (milder neutrophilic inflammation), cluster 2 (neutrophilic mixed with type 2 inflammation), cluster 3 (most severe neutrophilic inflammation), and cluster 4 (type 2 mixed with epithelial inflammation). Neutrophilic inflammation was present in all clusters; however, each cluster was associated with varying degrees of neutrophilic inflammation, a distinct microbiome profile and future exacerbation risk. The endotypes identified in clusters 2 and 3 were associated with increased risk of exacerbation during follow-up [135].

### Targeting inflammation in patients with bronchiectasis

The treatment of bronchiectasis is heavily focused on antibiotics, airway clearance and mucoactive medications and therefore primarily addresses the infection and mucociliary clearance components of the vicious vortex. These aspects of the disease are also the most intensively studied. Often, treatment approaches are empirical, such as the use of antibiotic therapy for the management of exacerbation and haemoptysis [6]. These management approaches do not address all aspects of the pathophysiology of bronchiectasis, particularly neutrophil-driven inflammation. In contrast to the other components of the vicious vortex in bronchiectasis, there are no proven direct anti-inflammatory treatments for this disease and this is a current unmet need for patients. Although anti-inflammatory agents such as inhaled corticosteroids or statins are widely used to treat bronchiectasis, they are not recommended [16]. Anti-IL-5 and anti-IL5Rα therapy may be beneficial for patients with bronchiectasis with an eosinophilic endotype [163].

Currently, macrolide antibiotics may be used to address inflammation in bronchiectasis. Macrolide antibiotics are known for their antimicrobial properties; however, they also have immunomodulatory effects. Small clinical studies have demonstrated that continuous treatment with macrolide antibiotics for 6–12 months in patients with bronchiectasis reduced the risk of exacerbations and their long-term use is recommended in patients with bronchiectasis with three or more exacerbations per year [16, 164–166]. However, the advantages of macrolide maintenance therapy must be balanced against potential risks, including emergence of bacterial and mycobacterial resistance, cardiotoxicity and ototoxicity [167].

Treatments that target NSPs represent a potential approach in the management of bronchiectasis that may help address the unmet need of treating neutrophil-driven inflammation. These mediators are pro-inflammatory and elevated levels correlate with clinical outcomes. In patients with bronchiectasis who have elevated sputum NE activity, A1AT supplementation is being evaluated to reduce airway inflammation and improve neutrophil function (Bronchiectasis Alpha-1 Augmentation Trial (BATMAN); NCT05582798) [168]. A1AT inhibits NE; however, NE and CatG can inactivate A1AT in bronchiectasis [49, 101]. Several molecules have been tested to directly target NE activity in bronchiectasis. In previous phase 2 trials, treatment with NE inhibitors AZD9668 and BAY 85–8501 led to improvements in FEV_1_; however, no significant changes were observed in QoL scores [169, 170]. Clinical trials have been conducted to assess the safety and efficacy of drugs that inhibit activation of NSPs, by targeting the protease that activates them, DPP1. As previously mentioned, patients with PLS lack DPP1, and therefore, the main symptoms of PLS, such as diffuse palmoplantar keratoderma and periodontitis leading to premature deciduous tooth loss, are of special interest in clinical trials of DPP1 inhibitors [171].

In the phase 2 WILLOW trial, approximately 250 patients with bronchiectasis were treated with the DPP1 inhibitor brensocatib 10 or 25 mg once daily or placebo once daily for 24 weeks. Compared with placebo, brensocatib treatment resulted in a significantly prolonged time to first exacerbation (primary end-point), a reduction in the annualised rate of exacerbations and a reduction in NSP activity. The proportion of patients who had an adverse event during the trial that led to discontinuation was similar across the brensocatib and placebo groups, with little evidence that discontinuation was related to treatment [171]. ASPEN, a phase 3, randomised, double-blind, placebo-controlled trial of brensocatib, is complete (ClinicalTrials.gov identifier: NCT04594369​). This trial assessed the efficacy and safety of brensocatib at 10 and 25 mg doses or placebo administered once daily for 52 weeks in a larger population of patients with bronchiectasis. The trial met its primary efficacy end-point, the rate of bronchiectasis exacerbations over the 52-week treatment period. The full publication of the results of the trial is expected in 2025.

A novel DPP1 inhibitor, BI 1291583, is also under investigation for the treatment of bronchiectasis. In a phase 1 characterisation study, BI 1291583 exhibited a good safety profile and was well tolerated in healthy volunteers [172]; results from a phase 2 study were recently published (ClinicalTrials.gov identifier: NCT05238675) [173].

## Conclusions

Bronchiectasis is an increasingly common chronic inflammatory lung disease that significantly impairs health status and QoL and places a heavy burden on both patients and healthcare systems. This review focuses on the key role of chronic neutrophilic inflammation in the pathogenesis of bronchiectasis *via* the release of NSPs, NETs and other toxic compounds. Neutrophils and their associated products contribute to all components of the vicious vortex in bronchiectasis. Excessive release of NSPs overwhelms the antiprotease response and promotes the protease–antiprotease imbalance observed in bronchiectasis, which correlates to inflammation, disease progression and pathogenesis. There is a strong and growing evidence base for the development of new management strategies that could improve symptoms and reduce exacerbations in patients with bronchiectasis, but there are no medications approved specifically for bronchiectasis, underscoring an unmet need for these patients. This review highlights novel therapies that are being investigated to target neutrophilic inflammation and how these may be beneficial. The identification of inflammation as a treatable trait may lead to more personalised and novel treatments in bronchiectasis.

**Questions for future research**

- How could bronchiectasis research contribute to a better understanding of neutrophilic inflammation?
    • How do neutrophils from patients with bronchiectasis function differently than those from healthy individuals?
    • Can anti-inflammatory therapy help decrease the use of short- or long-term antibiotics in patients with bronchiectasis?
- How can inflammatory endotype and phenotype data be translated into clinical practice?
    • How can patients be better stratified by endotype to guide precision medicine?
    • How does overlapping eosinophilic and neutrophilic inflammation affect treatment plans and treatment response?
- How can biomarkers of neutrophilic inflammation be developed and implemented?
    • How can biomarkers reliably differentiate between overlapping inflammatory endotypes?
    • How can inflammatory biomarkers be incorporated into clinical practice?
    • Can inflammatory biomarkers be used in the clinic to help predict treatment response?
